# Sit-stand and stand-sit transitions in older adults and patients with Parkinson’s disease: event detection based on motion sensors versus force plates

**DOI:** 10.1186/1743-0003-9-75

**Published:** 2012-10-07

**Authors:** Agnes Zijlstra, Martina Mancini, Ulrich Lindemann, Lorenzo Chiari, Wiebren Zijlstra

**Affiliations:** 1 Center for Human Movement Sciences, University of Groningen, University Medical Center Groningen, Antonius Deusinglaan 1, Groningen 9700 AD, The Netherlands; 2 Balance Disorders Laboratory, Department of Neurology, Oregon Health & Science University, Portland, OR, USA; 3 Klinik für Geriatrische Rehabilitation, Robert-Bosch-Krankenhaus, Stuttgart, Germany; 4 Department of Electronics, Computer Science & Systems, University of Bologna, Bologna, Italy

**Keywords:** Postural transitions, Body-fixed-sensor, Method comparison, Older adults, Parkinson’s disease, Independent-living

## Abstract

**Background:**

Motion sensors offer the possibility to obtain spatiotemporal measures of mobility-related activities such as sit-stand and stand-sit transitions. However, the application of new sensor-based methods for assessing sit-stand-sit performance requires the detection of crucial events such as seat on/off in the sensor-based data. Therefore, the aim of this study was to evaluate the agreement of detecting sit-stand and stand-sit events based on a novel body-fixed-sensor method with a force-plate based analysis.

**Methods:**

Twelve older adults and 10 patients with mild to moderate Parkinson’s disease with mean age of 70 years performed sit-stand-sit movements while trunk movements were measured with a sensor-unit at vertebrae L2-L4 and reaction forces were measured with separate force plates below the feet and chair. Movement onsets and ends were determined. In addition, seat off and seat on were determined based on forces acting on the chair. Data analysis focused on the agreement of the timing of sit-stand and stand-sit events as detected by the two methods.

**Results:**

For the start and end of standing-up, only small delays existed for the start of forward trunk rotation and end of backward trunk rotation compared to movement onset/end as detected in the force-plate data. The end of forward trunk rotation had a small and consistent delay compared to seat off, whereas during sitting-down, the end of forward trunk rotation occurred earlier in relation to seat on. In detecting the end of sitting-down, backward trunk rotation ended after reaching the minimum in the below-feet vertical force signal. Since only small time differences existed between the two methods for detecting the start of sitting-down, longer movement durations were found for the sensor-based method. Relative agreement between the two methods in assessing movement duration was high (i.e. ICCs ≥ 0.75), except for duration of standing-up in the Parkinson’s patients (ICC = 0.61).

**Conclusions:**

This study demonstrated high agreement of body-fixed-sensor based detection of sit-stand and stand-sit events with that based on force plates in older adults and patients with mild to moderate Parkinson’s disease. Further development and testing is needed to establish reliability for unstandardized performance in clinical and home settings.

## Background

The ability to safely perform mobility-related activities in daily life, such as rising from a chair and sitting down, is a prerequisite for maintaining independent functioning. Difficulties in performing mobility-related activities can lead to a less active lifestyle and a subsequent deterioration in overall functioning. There is evidence from epidemiological studies that many falls of older persons occur during transfer movements [1]. A fall during a rise from sit to stand can be related to the inability to counteract unexpected external forces, vestibular impairment, orthostatic hypotension, or to an age-related overall deterioration in neuromuscular functioning. Several features of sit-to-stand or stand-to-sit performance have been associated with falls or falls risk, e.g. transition duration and number of attempts in community-dwelling older subjects [2], rate of rise in force and postural sway in subjects post-stroke [3] and five-times-sit-to-stand time in patients with Parkinson’s disease [4]. Early identification of impaired sit-stand-sit transitioning and administration of tailored interventions may prevent loss of functional abilities and fall incidents. Easy applicable objective methods that can be used to assess or monitor the performance of sit-stand and stand-sit movements can assist in developing effective interventions and in optimizing individual application of interventions.

Previous studies using different laboratory techniques such as optoelectronic systems, electromyography and force plates have analyzed the sit-stand movement (see Galli et al. [5] for an overview of several studies). For evaluating sit-stand-sit ability in clinical and home settings, methods based on light-weight, wireless body-fixed sensors seem a good option [6]. Earlier studies have used motion sensors attached to trunk and/or leg segments for time detection and analyzing kinematics [2,7-10]. Most of these studies used a sensor on the upper trunk and focused only on the sit-to-stand transition. The present study evaluates a novel method which is based on a single, hybrid motion sensor incorporating accelerometers and gyroscopes attached to the lower back, and a new algorithm for detecting sit-stand as well as stand-sit events based on trunk rotation. Important advantages of a sensor location at the lower back are that the sensor can be worn over longer duration in a belt, and that data measured at this location can also be used for other analyses, i.e. assessment of gait parameters [11,12], objective quantification of functional mobility tests such as the Timed Up and Go test [13,14], activity detection [15,16] and possibly detection of falls [17].

Portable force plates can be used as an easy method to assess the timing of important sit-stand events in clinical and home settings [18]. Particularly the combination of separate force plates below the feet and chair [19] allows for an exact determination of seat-off and seat-on, and was therefore used in this study for a method comparison in investigating the newly developed, body-fixed-sensor (BFS) method for sit-stand and stand-sit timing detection. The aim of this study was to evaluate the agreement of event detection and duration estimation based on accelerations and angular velocities of the lower back with that based on ground reaction forces below the feet and chair during standardized sit-stand and stand-sit testing in older adults without specific age-related pathology as well as in Parkinson’s patients. Patients with Parkinson’s disease were included since they often display problems during sit-stand-sit transitions. Hence, their data were used to investigate whether the novel method yields similar results for event detection in older adults with and without difficulties in performing sit-stand-sit transitions.

## Methods

### Subjects and protocol

In this experimental study, the method comparison was performed in a group of 12 older adults who did not report any serious neurologic and/or musculoskeletal conditions and in a group of 10 patients with Parkinson’s disease (PD). Characteristics are summarized in Table 1. The severity of PD was graded from 2 to 3 according to the Hoehn and Yahr (H&Y) staging [20]. Patients did not report other disorders that seriously affect the neuro-musculoskeletal system. Study procedures were approved by the local Medical Ethical committee and all subjects signed an informed consent. 

**Table 1:** Subject characteristics

	**Older adults**	**Patients with PD**
Gender	6 M, 6 F	8 M, 2 F
Age (years)	70.3 (59–83)	70.0 (61–77)
Height (cm)	172.5 (163–187)	178.2 (159–191)
Weight (kg)	79.8 (62–102)	85 (67–105)

Mean, minimum and maximum values are indicated.

The patients with PD were tested when they were on anti-parkinsonian medication. A standard chair (seat height 47 cm, seat depth 45 cm) without backrest and with armrests was used. The average time score of three repetitions of the Timed Up and Go (TUG) test [21] was used to evaluate functional mobility. At the start of a sit-stand-sit movement cycle, the subject sat in the back of the seat and had both feet on the ground. Subjects were instructed to stand up from the chair, stand still for 3–5 seconds and sit down again. Each subject performed two sets of three trials. During the first set, subjects were free to use the armrests and during the second set subjects were instructed to cross their arms in front of the trunk.

### Data acquisition

Data acquisition included the measurement with 2 Bertec force plates (each sized 0.40 m x 0.60 m) by an Optotrak Data Acquisition Unit (Northern Digital Inc., Waterloo, Canada) and 3D motion data by a wireless hybrid, body-fixed sensor (DynaPort Hybrid, McRoberts BV, The Hague, NL). All data were measured at a sampling frequency of 100 Hz. One force plate was positioned below the chair and the other, on which the feet were resting, in front of the chair. The hybrid sensor (size 87 x 45 x 14 mm, weight 74 g) was inserted in an elastic belt and centered on the lower back at the level of vertebrae L2-L4. The sensor measured 3D-accelerations (± 2 g) and 3D-angular velocities (± 100 deg/s). Data were stored on a micro secure digital (SD) memory card that was inserted in the hybrid sensor. For synchronization, the two systems were cable-connected and a marker signifying the start of a trial was simultaneously set in the signals of both force plates and the hybrid sensor by pushing a button.

### Data analysis

**Figure 1:**
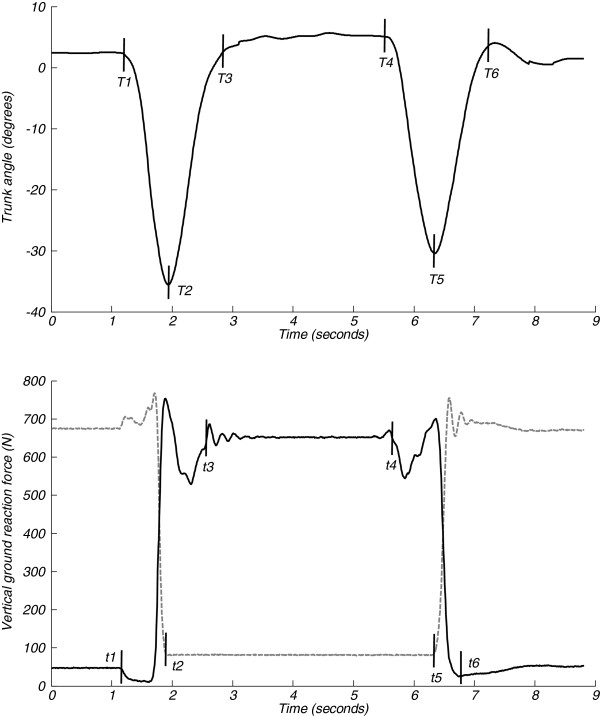
**Event detection in sit-stand-sit transitions. a** Trunk angle in the sagittal plane and events T1-T6 for a sit-stand-sit movement performed with arms crossed in front of the trunk by an older subject. **b** Vertical force signal below feet (black, solid line) and chair (grey, dashed line) and events t1-t6 for the same sit-stand-sit movement. See section ‘data analysis’ for a description of the events.

For the BFS data, signal processing and detection of sit-stand-sit events was performed using on-line software for blinded analysis as provided by the supplier (available as a module of the DynaPort MoveTest at http://www.mcroberts.nl). In the software, trunk angles in the sagittal plane were calculated using the acceleration and angular velocity data [22]. Vertical velocities were calculated based on the linear vertical accelerations that were obtained after removing the gravity component from the acceleration data by correcting for the orientation of the sensor, i.e. trunk angle. A positive or negative peak in the vertical velocity signal was used for identifying a standing-up or sitting-down movement. The timing of sit-stand and stand-sit events were detected based on the sine of the trunk angle after removing drift and noise from the signal using a discrete wavelet transformation [2]. Figure 1a illustrates the detection of events for the BFS method. *The start of standing-up (T1)* was defined as the end of the first plateau in the signal indicating start of forward trunk rotation. *Seat off (T2)* was assessed based on the first dip in the signal indicating end of forward trunk rotation. *The end of standing-up (T3)* was defined as the start of the plateau after the dip indicating the end of backward trunk rotation. Similarly as for standing-up, *the start (T4)*, and *end of sitting-down (T6)* were defined based on the plateaus before and after trunk rotation, and *seat on (T5)* was assessed based on the second dip indicating the end of forward trunk rotation. Plateaus were identified where the slope of the signal was smaller than 0.1. 

For the force-plate data, signal calibration and detection of sit-stand-sit events was performed using a Matlab (The Mathworks, Inc.) algorithm. The detection algorithm was based on the approach described by Lindemann et al. [18] and defined events based on the vertical force signals. Figure 1b illustrates the detection of events for the force-plate method:

*The start of standing-up (t1)* was defined as the point when the force beneath the feet decreased by more than 10% of feet weight.

*Seat off (t2)* was defined as the first point when the force beneath the chair was equal to chair weight.

*The end of standing-up (t3)* was defined as the first point when the force beneath the feet started to fluctuate around body weight. This point was searched for starting from the instant of maximum peak force above body weight, thereafter the force decreased below body weight until it again increased; the first point when the force was equal to body weight was taken as the end of standing-up. When armrests were used, a peak force above body weight was often absent. Therefore, in these cases, the end of standing-up was defined as the first instant after movement onset when the force beneath the feet was equal to body weight.

*The start of sitting-down (t4)* as the point when the force beneath the feet started to decrease by more than 1.5% of body weight.

*Seat on (t5)* was defined as the first point after the start of sitting-down when the force beneath the chair was higher than chair weight.

*The end of sitting-down (t6)* was defined as the point when the force beneath the feet reached its minimum after the instant of seat on.

For the BFS data, the sit-stand and stand-sit movement was divided into a flexion and extension phase based on direction of trunk rotation and maximum angular velocities for flexion and extension phases were obtained. For the force-plate data, the sit-stand and stand-sit movement was divided into a first phase from start to seat off/on and a second phase from seat off/on to end.

### Statistical analysis

The agreement between the BFS method and the force-plate method was expressed as single measures, two-way mixed, type consistency intra-class correlation coefficient (ICC_3,1_), associated 95% confidence interval (CI), and limit of agreement (LOA). An ICC_3,1_ of 0.75 or above was interpreted as high agreement as suggested by Burdock et al. [23]. LOAs for movement duration were calculated according to Bland and Altman [24] as 1.96 times the standard deviation of differences between the two methods. After Kolmogorov-Smirnov test approved normal distribution of data, parametric tests were applied. Paired samples *t*-tests were used to determine systematic differences between methods, statistical significance was set at *p* < 0.05. Individual trial data were used for ICCs, LOAs and paired *t*-tests. In comparing older subjects and patients with PD, the mean of all three trials was taken. Corresponding coefficients of variation (CoVs) were calculated as the standard deviation divided by the individual mean. Independent samples *t*-tests were used to determine systematic differences between groups. After applying a Bonferroni correction, the significance level was set at *p* < 0.0125.

## Results

In the patients with PD, two trials with free use of the armrests and one trial with arms crossed in front of the trunk were excluded from the analyses since the sit-stand-sit movement was not performed according to instructions. In 15 of the 36 trials (41.7%) when armrests were used during standing-up there was no peak vertical force above body weight to use as a starting point for detecting the end of standing-up. Therefore, in these cases, the end of standing-up was detected as the first point after movement onset when the force beneath the feet was equal to body weight.

### Agreement in assessing total duration

**Figure 2:**
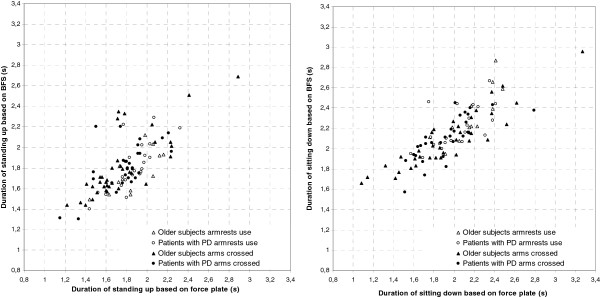
Movement durations estimated from body-fixed-sensor (BFS) data are plotted against those estimated from force-plate data.

**Figure 3:**
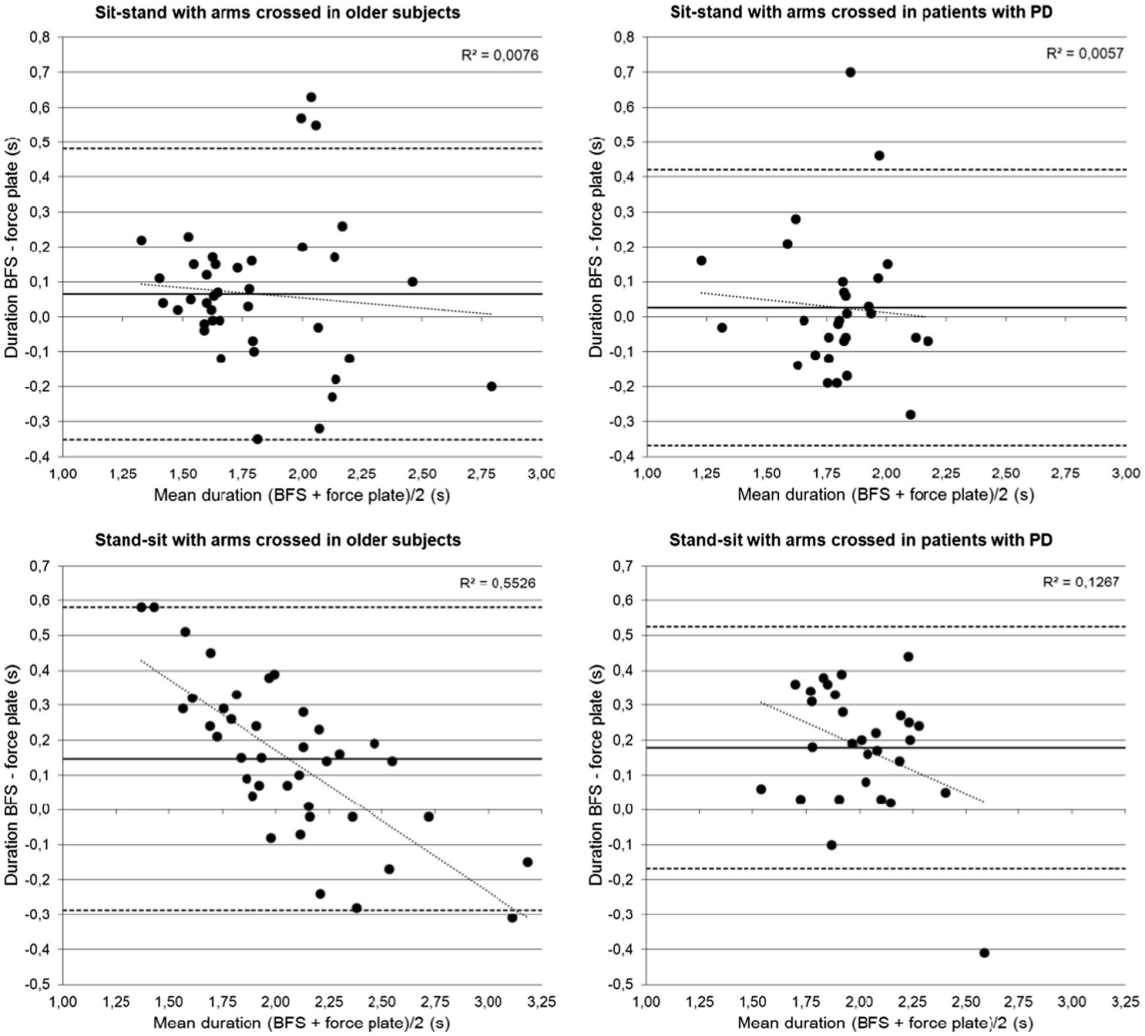
**Bland-Altman plots showing the differences in movement duration between the body-fixed-sensor (BFS) and force-plate method. a-d** A reference line (solid) for the mean of difference between the two methods, lines for plus (upper, dashed) and minus (lower, dashed) 1.96 times the standard deviation of differences, and a linear trend line with the coefficient of determination (R^2^) are given.

Individual sit-stand and stand-sit duration data for the BFS and force-plate method are illustrated in Figure 2 by scatter plots. Four out of 12 older subjects and 8 out of 10 patients with PD used the armrests when they were free to do so. In Table 2 mean duration data for performance with arms crossed in front of the trunk are presented and in Table 3 corresponding results for agreement are presented. ICCs_3,1_ were high (≥ 0.75) except for standing-up duration in the patients with PD. Estimated LOAs were 17 to 23% of the corresponding mean duration of the two methods (see Figure 3 for Bland-Altman plots). No significant differences between methods in standing-up duration existed whereas the BFS method yielded significantly longer sitting-down durations. The CoVs of sitting-down duration were smaller for the BFS method compared to the force-plate method in both the older subjects (*t* = −2.345, *p* = .039) and patients with PD (*t* = −2.508, *p* = .033). No differences between methods were found for the CoVs of standing-up duration (*t* = 0.145, *p* = .888; *t* = 0.356, *p* = .730). In contrast, when armrests were used the BFS method yielded shorter standing-up durations than the force-plate method in both the 4 older subjects (1.75 versus 1.86 seconds, 12 trials) and the 8 patients with PD (1.82 versus 1.87 seconds, 22 trials). The between-method difference in duration was significant for the total group of 12 subjects (*t* = −2.624, *p* = .013).

**Table 2:** Durations and maximum angular velocities in standing-up (SU) and sitting-down (SD)

		**Older subjects (n = 12)**		**Patients with PD (n = 10)**			
		**Duration [s]**	**CoV [%]**	**Duration [s]**	**CoV [%]**	***t *duration, CoV**	***p***
**BFS**	SU	**1.81** (0.26)	**8.05** (6.83)	**1.82** (0.16)	**9.44** (6.97)	−0.096	.925
		*1.49-2.25*	*1.23-17.86*	*1.47-2.01*	*2.13-19.78*	−0.474	.641
	SD	**2.09** (0.25)	**6.99** (3.32)	**2.10** (0.18)	**6.76** (3.21)	−0.171	.866
		*1.74-2.57*	*2.83-13.29*	*1.71-2.33*	*2.40-12.91*	0.167	.869
**Force plate**	SU	**1.75** (0.31)	**7.77** (4.80)	**1.79** (0.19)	**8.78** (7.13)	−0.323	.750
		*1.36-2.45*	*0.96-15.42*	*1.43-1.99*	*0.60-23.86*	−0.397	.696
	SD	**1.93** (0.40)	**10.67** (5.39)	**1.92** (0.19)	**10.29** (6.98)	0.071	.944
		*1.18-2.69*	*0.98-20.58*	*1.68-2.21*	*2.41-25.06*	0.143	.888
		**Duration [s]**	**ωmax [deg/s]**	**Duration [s]**	**ωmax [deg/s]**	***t *duration, ωmax**	***p***
**BFS SU**	Flexion	**0.86** (0.15)	**99.44** (13.04)	**0.83** (0.07)	**102.26** (17.85)	0.718	.483
		*0.68-1.16*	*73.27-116.72*	*0.67-0.91*	*77.00-133.44*	−0.428	.673
	Extension	**0.95** (0.14)	**85.10** (16.92)	**0.99** (0.10)	**68.70** (6.31)	−0.853	.404
		*0.76-1.24*	*59.99-111.83*	*0.80-1.15*	*59.87-79.18*	3.108	.007*
**BFS SD**	Flexion	**0.96** (0.13)	**93.85** (15.36)	**1.06** (0.17)	**66.13** (19.96)	−1.603	.125
		*0.79-1.19*	*67.63-124.16*	*0.75-1.23*	*37.45-101.63*	3.683	.001*
	Extension	**1.13** (0.18)	**80.79** (13.21)	**1.04** (0.11)	**83.65** (22.56)	1.335	.197
		*0.90-1.49*	*59.42-102.37*	*0.88-1.22*	*53.94-119.86*	−0.370	.716

Averaged total durations and corresponding coefficients of variation (CoVs) for the condition with arms crossed in front of the trunk as determined from body-fixed-sensor (BFS) and force-plate data, as well as averaged durations and maximum angular velocities (ωmax) of flexion and extension phases. Mean, standard deviation, minimum and maximum is indicated.

***t***: two-tailed independent samples *t*-test, * significant difference.

**Table 3:** Agreement for body-fixed-sensor and force-plate method in duration estimation of standing-up (SU) and sitting-down (SD)

	**Older subjects (36 trials)**				**Patients with PD (29 trials)**			
	**ICC**_**3,1**_	**LOA (s)**	***t***	***p***	**ICC**_**3,1**_	**LOA (s)**	***t***	***p***
	**(95% CI)**				**(95% CI)**			
SU	**0.784**	0.417	1.725	.093	**0.612**	0.394	0.702	.488
	(0.616-0.884)				(0.322-0.797)			
SD	**0.821**	0.433	4.259	.000*	**0.747**	0.346	5.467	.000*
	(0.676-0.904)				(0.528-0.873)			

Intra-class correlation coefficients (ICCs_3,1_), corresponding 95% confidence intervals (CIs), limits of agreement (LOAs) and paired samples *t*-test results for the condition with arms crossed in front of the trunk.

***t***: two-tailed paired samples *t*-test, * significant difference.

### Comparison of sit-stand-sit events and phase durations

**Figure 4:**
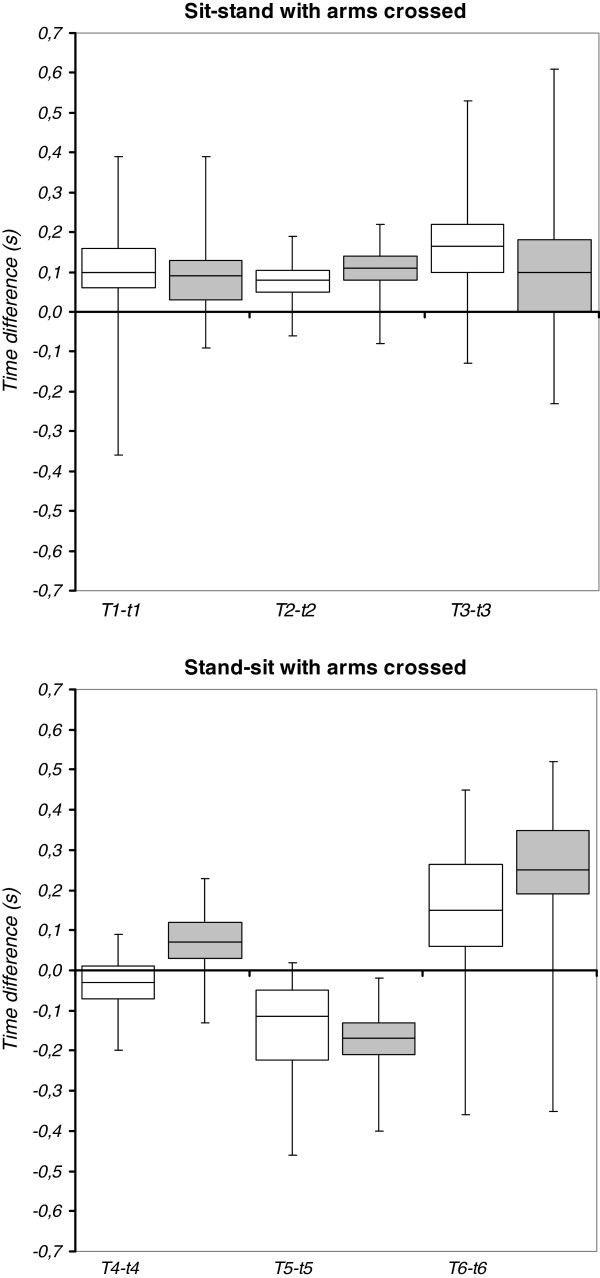
**Time differences between body-fixed-sensor (T) and force-plate (t) method for detecting sit-stand-sit events (1–6). a-b** Older subjects (white boxes), patients with PD (grey boxes). Boxes denote median value, first and third quartile and whiskers denote minimum and maximum value.

Time differences between the BFS method (T) and force-plate method (t) in detecting events of sit-stand-sit performed with arms crossed are illustrated in Figure 4 by box plots. Median values of time differences were similar (ranging from 0.09 to 0.17 seconds) for start (T1-t1) and end (T3-t3) of standing-up. For the end of forward trunk rotation in standing-up (T2) as compared to the instant of seat off (t2) median values of time differences were even smaller (0.08 and 0.11 seconds). Consequently, for the flexion phase duration in standing-up (T2-T1) compared to the duration to seat off (t2-t1) no significant difference existed for both the older subjects (mean 0.86 versus 0.88 seconds, *t* = −0.725, *p* = .473) and patients with PD (mean 0.82 versus 0.81 seconds, *t* = 0.546, *p* = .589). A significant difference for extension phase duration (T3-T2) compared to duration from seat off to end (t3-t2) did exist in the older subjects (mean 0.95 versus 0.87 seconds, *t* = 3.879, *p* = .000), but did not exist in the patients with PD (mean 0.99 versus 0.98 seconds, *t* = 0.379, *p* = .707). There was a moderate agreement between methods in the duration of the first phase (ICC_3,1_ = 0.71, 95%CI = 0.50-0.84) and second phase (ICC_3,1_ = 0.67, 95%CI = 0.45-0.82) of standing-up in the older subjects. The agreement was low in the patients with PD for both first (ICC_3,1_ = 0.41, 95%CI = 0.06-0.67) and second phase duration (ICC_3,1_ = 0.38, 95%CI = 0.02-0.65). For sitting-down, median values of time differences between the two methods were not similar (ranging from −0.17 to 0.25 seconds) over the three events.

### Group comparison

The mean TUG test score was 12.3 s (range 9.4-14.8 s) for the older subjects and 12.1 s (range 10.4-15.3 s) for the patients with PD (*t* = 0.343, *p* = .735). Significant between-group differences existed only for maximum angular velocity during the extension phase of standing-up and flexion phase of sitting-down (Table 2). Time differences between methods in detected sit-stand-sit events for the arms-crossed condition (Figure 4) did not differ between groups for standing-up (T1-t1: *t* = 0.302, *p* = .764; T2-t2: *t* = −1.556, *p* = .125; T3-t3: *t* = 1.217, *p* = .228) whereas significant differences were observed between groups for the start of sitting-down (T4-t4: *t* = −5.273, *p* = .000; T5-t5: *t* = 1.417, *p* = .161; T6-t6: *t* = −2.498, *p* = .015).

## Discussion

The aim of this study was to evaluate the agreement of event detection and duration estimation in standardized sit-stand and stand-sit transitions based on i) accelerations and angular velocities of the lower back as collected by a body-fixed-sensor unit and ii) ground reaction forces below the feet and the chair. To the best of our knowledge, the present study was the first to evaluate BFS based events of standing-up as well as sitting-down and to perform a gold-standard comparison of BFS based event instants to the instants of seat off and seat on as accurately determined by a force plate below the chair.

In the group of older subjects, high relative agreement between the two methods, as determined by intra-class correlation, for assessing total duration of sit-stand and stand-sit transitions was found. The relative agreement between the two methods was somewhat less in the group of patients with Parkinson’s disease, i.e. agreement was moderate for stand-sit duration and high for stand-sit duration. There were no systematic differences between the two methods in assessed duration or variability in duration of standing-up, other than an underestimation for the BFS method in the duration of standing-up when armrests were actually used. Two factors may have contributed to the underestimation. First, when armrests are used, less forward trunk rotation is needed in preparation for rising and as a consequence the end of backward trunk rotation may occur earlier in the sequence of events than when armrests are not used. Second, due to more controlled movement in using armrests less stabilizing trunk movements might be needed to keep balance after rising. The BFS method overestimated stand-sit duration compared to the force-plate method and as a consequence the BFS method underestimated the variability as percentage of stand-sit duration. The systematic difference in stand-sit duration between the two methods may be explained by backward trunk rotation occurring after the shift of body weight to the chair.

The start as well as end of standing-up was detected with a small delay for the BFS method as compared to the force-plate method. Sit-stand initiation may cause the vertical ground reaction force below the feet to decrease before there is any trunk rotation. Since the end of sit-stand was determined by the force-plate method before stabilization during stance, trunk rotation to keep balance after extending to stance may have caused the small delay in end detection for the BFS method. Only a small systematic delay of approximately 0.1 seconds existed for the end of forward trunk rotation in standing-up as compared to the instant of seat off as accurately determined by a force plate below the chair. Thus, the end of forward trunk rotation can be used to identify seat-off, which allows a specific analysis of the subsequent rising phase. There were no systematic differences in phase durations between the two methods, other than a small systematic difference between extension phase duration and duration from seat off to end in the group of older subjects. The relative agreement between the two methods for phase durations was not high, presumably because the differences in phase duration between the two methods were relatively large for the short phase durations. The start of sitting-down was detected with a small delay for the BFS method as compared to the force-plate method, which was specifically the case for the group of patients with PD. The instant of decrease in vertical ground reaction force below the feet may occur before initiation of forward trunk rotation. The end of sitting-down was detected with a marked systematic time difference between the two methods, which resulted in the overestimation of sitting-down duration by the BFS method. Furthermore, the end of forward trunk rotation in sitting-down occurred earlier in relation to the instant of seat on as accurately determined by a force plate below the chair.

Between-group differences were largely absent. This may be because patients were on medication to minimize the symptoms of PD and most patients had only mild disease with or without some postural instability (H&Y stage 2 and 2.5). Furthermore, only three patients reported having difficulties in rising from a chair during daily-life, and the total number of falls in the six months prior to the experiment was low, i.e. three due to tripping over floor obstacles, three that occurred in cycling and one in rising from a chair. TUG-test time scores indicated similar functional mobility for the patients and older adults. Possibly, performance times do not have sufficient discriminative ability as is supported by Zampieri et al. [25] who demonstrated that the time to complete the TUG test is not sensitive to differentiate between early, untreated PD and healthy controls.

For investigating whether the novel BFS method yields adequate results for timing detection in older persons with impaired transitioning, the patients with PD that were included in this study may not have been entirely suitable since only some had significant problems in rising to stand. The agreement of events detected by the BFS method to those detected by the force-plate method in patients that do demonstrate significant difficulties in performing sit-stand and stand-sit tasks remains to be investigated. Some caution is recommended in generalizing the results of the study. The high agreement in sit-stand-sit events between the two methods was observed for single movement performance with arms crossed in front of the trunk and when starting from a standardized position. The method needs to be further tested under a variety of task conditions in order to evaluate the timing detection for clinical application.

Although the BFS method did not demonstrate differences in timing aspects of sit-stand-sit transitioning between the patients with mild PD and healthy older adults, the BFS method can be useful for application in patients in which medication does not lead to an optimal reduction of motor symptoms. It should be noted that, besides detecting the timing of events, the BFS method can be useful in quantifying specific aspects of movement phases such as peak angular velocity in trunk flexion/extension and power in rising to stance [10]. In this study, between-group differences in maximum trunk angular velocity during the extension phase of standing-up and the flexion phase of sitting-down were found, implying that the patients with PD had a different movement strategy as compared to the older adults. For evaluating performance in daily-life circumstances, the BFS method can therefore be a relevant addition to stopwatch-timed tests.

## Conclusions

This study demonstrated high agreement of detecting standing-up events based on a novel method using a body-fixed-sensor unit at the lower back with that based on separate force plates below the feet and chair in older adults and patients with mild to moderate Parkinson’s disease. Our results indicate that the end of forward trunk rotation can be used to identify seat-off. Systematic between-method differences were demonstrated for detecting sitting-down events, however relative agreement for movement duration was high. Although the instant of seat-on cannot be determined as well as with the combination of two force plates, the body-fixed-sensor method can be recommended because unlike the force-plate method, it provides information about trunk rotation, which is an important indicator of differences in sit-stand-sit performance. Further development and testing is needed for reliable detection of unstandardized sit-stand and stand-sit transitions in varying real-life circumstances.

## Abbreviations

BFS: Body-fixed-sensor; CI: Confidence interval; CoVs: Coefficients of variation; H&Y: Hoehn and Yahr; ICC: Intra-class correlation coefficient; LOA: Limit of agreement; PD: Parkinson’s disease; TUG: Timed up and go.

## Competing interests

The authors declare that they have no competing interests.

## Authors’ contributions

AZ participated in the design of the study, the acquisition and analysis of data, and drafted the manuscript. WZ conceived the study, participated in its design and in the interpretation of data, and helped to draft the manuscript. MM, UL, and LC were involved in revising the manuscript. All authors read and approved the final manuscript.

## References

[B1] Rapp K Becker C Cameron ID Konig HH Buchele G Epidemiology of falls in residential aged care: analysis of more than 70,000 falls from residents of bavarian nursing homes J Am Med Dir Assoc 2012 13 187.e1 6 10.1016/j.jamda.2011.06.011 21816682

[B2] Najafi B Aminian K Loew F Blanc Y Robert PA Measurement of stand-sit and sit-stand transitions using a miniature gyroscope and its application in fall risk evaluation in the elderly IEEE Trans Biomed Eng 2002 49 843 851 10.1109/TBME.2002.800763 12148823

[B3] Cheng PT Liaw MY Wong MK Tang FT Lee MY Lin PS The sit-to-stand movement in stroke patients and its correlation with falling Arch Phys Med Rehabil 1998 79 1043 1046 10.1016/S0003-9993(98)90168-X 9749681

[B4] Mak MK Pang MY Parkinsonian single fallers versus recurrent fallers: different fall characteristics and clinical features J Neurol 2010 257 1543 1551 10.1007/s00415-010-5573-9 20449601

[B5] Galli M Cimolin V Crivellini M Campanini I Quantitative analysis of sit to stand movement: experimental set-up definition and application to healthy and hemiplegic adults Gait Posture 2008 28 80 85 10.1016/j.gaitpost.2007.10.003 18618956

[B6] Zijlstra W Aminian K Mobility assessment in older people: new possibilities and challenges Eur J Ageing 2007 4 3 12 10.1007/s10433-007-0041-9 PMC5546360 28794767

[B7] Janssen WG Bussmann JB Horemans HL Stam HJ Analysis and decomposition of accelerometric signals of trunk and thigh obtained during the sit-to-stand movement Med Biol Eng Comput 2005 43 265 272 10.1007/BF02345965 15865138

[B8] Boonstra MC van der Slikke RM Keijsers NL van Lummel RC de Waal Malefijt MC Verdonschot N The accuracy of measuring the kinematics of rising from a chair with accelerometers and gyroscopes J Biomech 2006 39 354 358 10.1016/j.jbiomech.2004.11.021 16321638

[B9] Giansanti D Maccioni G Benvenuti F Macellari V Inertial measurement units furnish accurate trunk trajectory reconstruction of the sit-to-stand manoeuvre in healthy subjects Med Biol Eng Comput 2007 45 969 976 10.1007/s11517-007-0224-8 17653580

[B10] Zijlstra W Bisseling RW Schlumbohm S Baldus H A body-fixed-sensor-based analysis of power during sit-to-stand movements Gait Posture 2010 31 272 278 10.1016/j.gaitpost.2009.11.003 19963386

[B11] Zijlstra W Assessment of spatio-temporal parameters during unconstrained walking Eur J Appl Physiol 2004 92 39 44 10.1007/s00421-004-1041-5 14985994

[B12] Dijkstra B Zijlstra W Scherder E Kamsma Y Detection of walking periods and number of steps in older adults and patients with Parkinson’s disease: accuracy of a pedometer and an accelerometry-based method Age Ageing 2008 37 436 441 10.1093/ageing/afn097 18487266

[B13] Weiss A Herman T Plotnik M Brozgol M Maidan I Giladi N Gurevich T Hausdorff JM Can an accelerometer enhance the utility of the Timed Up & Go Test when evaluating patients with Parkinson’s disease? Med Eng Phys 2010 32 119 125 10.1016/j.medengphy.2009.10.015 19942472

[B14] Weiss A Herman T Plotnik M Brozgol M Giladi N Hausdorff JM An instrumented timed up and go: the added value of an accelerometer for identifying fall risk in idiopathic fallers Physiol Meas 2011 32 2003 2018 10.1088/0967-3334/32/12/009 22094550

[B15] Dijkstra B Kamsma Y Zijlstra W Detection of gait and postures using a miniaturised triaxial accelerometer-based system: accuracy in community-dwelling older adults Age Ageing 2010 39 259 262 20083616 10.1093/ageing/afp249

[B16] Dijkstra B Kamsma YP Zijlstra W Detection of gait and postures using a miniaturized triaxial accelerometer-based system: accuracy in patients with mild to moderate Parkinson’s disease Arch Phys Med Rehabil 2010 91 1272 1277 10.1016/j.apmr.2010.05.004 20684910

[B17] Klenk J Becker C Lieken F Nicolai S Maetzler W Alt W Zijlstra W Hausdorff JM van Lummel RC Chiari L Lindemann U Comparison of acceleration signals of simulated and real-world backward falls Med Eng Phys 2011 33 368 373 10.1016/j.medengphy.2010.11.003 21123104

[B18] Lindemann U Claus H Stuber M Augat P Muche R Nikolaus T Becker C Measuring power during the sit-to-stand transfer Eur J Appl Physiol 2003 89 466 470 10.1007/s00421-003-0837-z 12712354

[B19] Chang CS Leung CY Liou JJ Tsai WW Evaluation of key points in the sit-to-stand movement using two force platforms Percept Mot Skills 2010 111 496 502 10.2466/10.15.26.PMS.111.5.496-502 21162451

[B20] Hoehn MM Yahr MD Parkinsonism - Onset progression and mortality Neurology 1967 17 427 10.1212/WNL.17.5.427 6067254

[B21] Podsiadlo D Richardson S The Timed Up and Go - A test of basic functional mobility for frail elderly persons J Am Geriatr Soc 1991 39 142 148 1991946 10.1111/j.1532-5415.1991.tb01616.x

[B22] Williamson R Andrews BJ Detecting absolute human knee angle and angular velocity using accelerometers and rate gyroscopes Med Biol Eng Comput 2001 39 294 302 10.1007/BF02345283 11465883

[B23] Burdock EI Fleiss JL Hardesty AS A new view of inter-observer agreement Person Psychol 1963 16 373 384 10.1111/j.1744-6570.1963.tb01283.x

[B24] Bland JM Altman DG Statistical methods for assessing agreement between 2 methods of clinical measurement Lancet 1986 1 307 310 2868172

[B25] Zampieri C Salarian A Carlson-Kuhta P Aminian K Nutt JG Horak FB The instrumented timed up and go test: potential outcome measure for disease modifying therapies in Parkinson’s disease J Neurol Neurosur Ps 2010 81 171 176 10.1136/jnnp.2009.173740 PMC3065923 19726406

